# North African dust intrusions and increased risk of respiratory diseases in Southern Portugal

**DOI:** 10.1007/s00484-021-02132-x

**Published:** 2021-04-22

**Authors:** T. Silva, M. Fragoso, R. Almendra, J. Vasconcelos, A. Lopes, A. Faleh

**Affiliations:** 1 University of Lisbon, Institute of Geography and Spatial Planning (IGOT), Centre of Geographical Studies (CEG), Lisbon, Portugal; 2 Department of Geography and Tourism, University of Coimbra, Centre of Studies in Geography and Spatial Planning (CEGOT), Coimbra, Portugal; 3 Polytechnic of Leiria , Leiria, Portugal; 4 Sidi Mohammed Ben Abdellah University-Fès , Fès, Morocco; 5 Portuguese Institute for Sea and Atmosphere (IPMA) , Lisbon, Portugal

**Keywords:** Portugal, North African dust, Respiratory diseases, Hospital admissions, DLNM

## Abstract

The study of dust intrusions in Portugal is still a subject on which little investigation has been made, especially in terms of their effects. Thus, this work aims to achieve two goals: firstly, to characterize the dust intrusions in the study area; and secondly, to evaluate the possible statistical association between the dust intrusion days and hospital admissions due to respiratory diseases. Dust intrusions in Portugal are prevalent during the summer season. During this season, the dust plumes tend to cover broader areas than in the other seasons and they have origin in the North African countries. In the study area for the period between 2005 and 2015, the relative risk of urgent hospitalizations due to respiratory diseases was 12.6% higher during dust intrusion days. In order to obtain this statistical association, a Distributed Lag Nonlinear Model was developed. With this work, we expect to help the development of further studies regarding North African dust intrusions in Portugal, more precisely their effects on human health.

**Supplementary Information:**

The online version contains supplementary material available at 10.1007/s00484-021-02132-x.

## Introduction

Desert dust intrusions are a growing study object across the world mainly because of their potential environmental and human health impacts. Thus, in Europe, especially in the southern countries, they are increasingly being studied by the scientific community.

Dust storms are events generated by the action of the wind which will cause the movement of sediments from the surface to the lower atmosphere (UNEP et al. 2016). Dust storms are the form of atmospheric pollution that affect people mostly (UNEP et al. 2016), not only because of the fine particle size that can be breathed into the lungs and invade the smallest airways, including alveolar tissue (Goudie 2014), but also because of other components as allergens, toxic chemicals (Goudie and Middleton 2006), microbes and fungi (Griffin et al. 2002).

Several papers have highlighted that particulate matter may trigger inflammatory stress responses associated with chronic obstructive pulmonary disease (Middleton 2017), asthma, bronchitis (Middleton 2017; Samoli et al. 2011a; UNEP et al. 2016), pneumonia, allergic rhinitis, irritations and inflammations in the upper respiratory system (UNEP et al. 2016) and cardiovascular conditions (Middleton et al. 2008). These diseases, along with flu, irritations and inflammations in the lower respiratory system, rhinitis and pneumoconiosis, have been chosen for this study.

Epidemiological studies have shown a relation between these dust intrusions and hospital admissions (Alessandrini et al. 2013; Brunekreef and Forsberg 2005; Chien et al. 2012; Gyan et al. 2005; Middleton et al. 2008) and mortality (Alessandrini et al. 2013; Mallone et al. 2011; Neophytou et al. 2013; Pérez et al. 2008, 2012; Sajani et al. 2011; Samoli et al. 2011b). Studies conducted by Alessandrini et al. (2013), Sajani et al. (2011) and Samoli et al. (2011a) have also shown a relation between these phenomena and the increase of respiratory diseases. Some of these studies evaluate these relations through lagged effect models, for example, Jiménez et al. (2010), Middleton et al. (2008) and Samoli et al. (2011a, 2011b). In the Iberian Peninsula, Linares et al. (2020), Ortiz et al. (2017) and Stafoggia et al. (2013, 2016) have shown that there is a relationship between exposure to particulate matter and an increase in mortality and hospital admissions regarding cardiovascular and respiratory disorders.

Other authors have studied different aspects of the dust intrusions like Escudero et al. (2005), Goudie and Middleton, (2006) and Griffin et al. (2002) who studied the dust chemical and organic composition; Couto et al. (2021), Russo et al. (2020), Díaz et al. (2017) and Salvador et al. (2013) who focused on the synoptic conditions during dust intrusion days; and more recently, Sousa et al. (2019) who studied the relation between dust intrusions days and heat waves. Several authors have applied Distributed Lag Nonlinear Regression Models to study the relationship between physical environmental health determinants (e.g., dust concentrations and meteorological parameters, among others) and their lagged effects on human health (Almendra et al. 2019a; Almendra et al. 2019b; Gasparrini and Armstrong 2011; Stafoggia et al. 2013; Silva 2015; Vasconcelos et al. 2013).

The association between dust events and hospitalizations or hospital morbidity is still not clear in Portugal, and studies are still very scarce, and therefore, it is an innovative study in the country. Thus, in this study, the association between hospital admissions due to respiratory diseases and the occurrence of dust intrusions in Central Alentejo (Portugal) was assessed.

Therefore, two main objectives have been established for the present study: first to characterize the North African dust intrusions in the study area, in terms of the temporal and spatial distribution, and the effects of particle concentrations on the air quality at the surface; and second, to evaluate the possible statistical association between the identified dust days and the emergency hospital admissions due to respiratory diseases.

## Study area

**Fig. 1:**
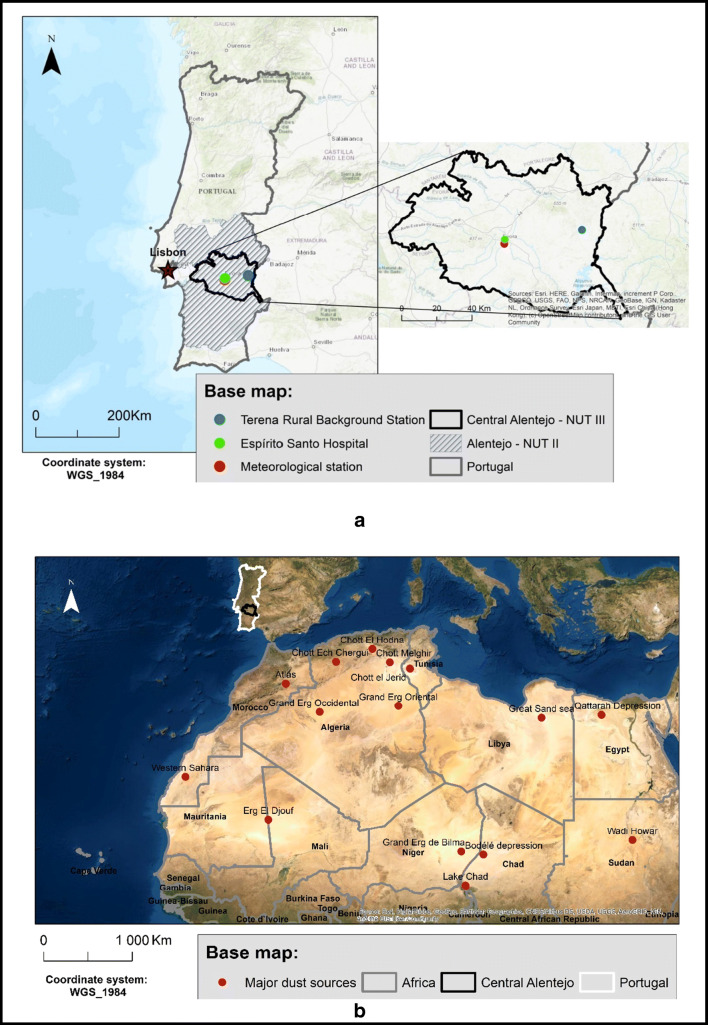
**S**tudy area (**a**) and North African major dust sources (**b**). The study area is also represented by the rural background station where the pollutants were measured and the hospital which supplied the internment data

Central Alentejo, NUT III, the central area of Alentejo region, is one of the statistical regions of Portugal, located in the south of the country, matching Évora district (Fig. 1). It has an area of 7393 km^2^ (8% of the Portuguese territory) (PIAAC-AC. 2018). Portugal, especially the study area, is located geographically close to North Africa and the Sahara Desert, hence the importance of this study. Central Alentejo had a mean population density of 20.9 inhabitants per square kilometre in 2018 with circa 154,500 residents (INE 2018). Of the 154,536 inhabitants, 26,671 are between 0 and 19 years old and 39,808 are 65 plus years old (INE 2018). The study area has one central public, Hospital do Espírito Santo (Fig. 1). Alentejo climate is classified, according to Köppen classification, as Csa (PIAAC-AC. 2018). The mean annual temperature is 16°C. During winter, temperature may reach as low as 5°C, and during the summer, it reaches temperatures higher than 30°C.

This study area has been chosen for two reasons: firstly and more importantly due to the availability of hospital admission data for Central Alentejo; and secondly, the region of Alentejo (NUT II) is one of the closest Portuguese regions to North Africa, and because of that, it is also one of the most affected by North African dust intrusions according to the air quality reports produced by the Portuguese Environmental Agency (for further information see the reports *identificação e avaliação de eventos naturais em Portugal no ano de… from 2006 to 2015)*.

## Data and methods

The APA (Agência Portuguesa do Ambiente—Portuguese Environmental Agency) reports about natural events detected in Portugal such as dust intrusions were crucial to this study because it enabled us to identify which days have been affected by North African dust. Between 2006 and 2015, 825 dust intrusion days were identified in Alentejo. To verify among these 825 intrusion days which dust intrusions overlapped the Central Alentejo region (Fig. 1), a checking procedure was carried out. Hence, we have strained the APA inventory into a study inventory (resulting in 246 days) using satellite imagery, backward trajectories and PM10 concentrations. There is no APA report for the year 2005, so the days were identified with the same methodology used to create the study inventory. The study inventory was also used as a mean of comparison for the Distribution Lag Nonlinear Regression Model used in the present study.

### Data

To reduce the size of APA inventory and to achieve the first objective, the following data was used:

- *APA inventories*: the criteria used by APA to identify a dust intrusion day is established by the European Commission in the document “Establishing guidelines for demonstration and subtraction of exceedances attributable to natural sources under the Directive 2008/50/EC on ambient air quality and cleaner air for Europe”. Among the five criteria established in this document, APA only uses three of the required measures (Table 1).
- *Satellite images:* real colour images and two aerosol concentration layers (deep blue aerosol optical depth and aerosol optical depth) from Terra and Aqua satellites with MODIS sensor (Table 1) were used to create the new inventory and to delimit the boundaries of the dust plumes.
- *HYSPLIT backward trajectories* proposed by Stein et al. (2015*)*: the HYSPLIT model was used to help reduce the APA inventory and to help understand which North African regions the dust came from, illustrating the pathway taken by the transported dust until they arrived in Central Alentejo. To build these models, some parameters had to be defined, such as the destination point (Table 1) and the atmospheric database (Reanalysis NCEP/NCAR). The backward trajectories were calculated always up to 5 days prior to the event (dust intrusion in Central Alentejo) for 700, 1500 and 2500 m of altitude (above ground level).
- *PM10 and PM2.5 daily concentrations*: the pollutants constituted another type of data that helped to strain the APA inventory. The daily mean limits defined by the European Commission were considered for both pollutants (Table 1). These data were retrieved from QualAr website (APA), for Terena rural station (Fig, 1). Only days with more than 12-h PM10/PM2.5 measures were taken into account to calculate the daily average.

**Table 1:** Metadata

Data	Period	Frequency	Spatial resolution	Source	Other characteristics
APA reports	2006–2015	Yearly	NUTS II-Alentejo	Agência Portuguesa do Ambiente (APA)	Criteria used by APA to identify dust intrusions: HYSPLIT models; PM10 and PM2.5 concentrations; dust intrusion prediction models (e.g., SKIRON, BSC-DREAM)
Satellite imagery—real colour	2005–2015	Daily	5 km pixel	NASA WorldView-Terra/MODIS	
Satellite imagery—aerosol optical depth	2005–2015	Daily	2 km pixel	NASA WorldView-Terra and Aqua/MODIS	Used to identify dust over oceans and dark land such as vegetated areas
Satellite imagery—deep blue aerosol optical depth	2005–2015	Daily	2 km pixel	NASA WorldView-Terra and Aqua/MODIS	Used to identify dust over deserts and arid lands
HYSPLIT models	2005–2015	Daily	North Africa/Iberian Peninsula	NOAA Air Resources Laboratory	Destination point defined: 38°34′ 47″ N, 7°54′36″ W
PM10 and PM2.5	2005–2015	Hourly	Terena	APA-QualAr	Missing values: PM10: 21%; PM2.5: 18%. Daily mean limits: PM10: 50 μg/m^3^; PM2.5: 25 μg/m^3^. Station location: 38°36′54″ N, 7°23′51″ W
Meteorological data	2005–2015	Daily	Évora/Geophysical centre	ICT-*Atmospheric Sciences Water and Climate;* KNMI Climate Explorer	Missing values: MeanT: 1.9%; MinT: 2.2%; MaxT: 1.9%; RH: 1.8%; WV: 1.6%; P: 1.6%. Meteorological station location: 38°31′48″ N, 7°54′36″ W
Hospital admissions data	2005–2015	Daily	NUTS III-Central Alentejo	Grouping of Health Centers in Central Alentejo.	ICD-9-CM.

*To achieve the second goal, the following data were also used:*

- *Meteorological data*: the used data were as follows: mean temperature (°C), maximum temperature (°C), minimum temperature (°C), wind velocity (m/s), relative humidity (%) and precipitation (mm), from Évora/Geophysical centre meteorological station (Table 1). These meteorological variables were important for two reasons: firstly, to assess the atmospheric and environmental conditions during dust intrusion days and to compare these same conditions between days with dust (APA and study inventories) and days without dust intrusions; and secondly, to be introduced in the DLNM as co-variables.
- *Hospital admissions data*: daily urgent hospitalizations due to respiratory pathologies (ICD9: 460-519—for more details see Table S1) at the *Espiríto Santo* hospital in Évora were taken into account (Table 1). The data was stratified by age group (0–16; ≥65) and season (from winter solstice to summer solstice).

### Methods

#### Straining the APA inventory

Days included in the study inventory had to fulfil two out of three criteria: (i) North African dust plume affecting the study area (whether visible by real colour satellite images or aerosol optical depth layers) had to be noticeable; (ii) the HYSPLIT backward trajectories had to prove that the air masses carrying the dust affecting the study area came from North Africa 5 days prior to the event registered in Central Alentejo; (iii) daily mean concentration of particulate matter PM10 and PM2.5 had to surpass the established daily mean limits of 50 μg/m^3^ for PM10 and 25 μg/m^3^ for PM2.5.

#### Statistical analysis and DLNM modelling

In an earlier stage of the research, some exploratory analyses were developed. One of these focused on the temporal distribution of the events (annual, seasonal and monthly). Seasons were defined according to the solstices of March and September and equinoxes of June and December. Also, the statistical characterization of the PM2.5 and PM10 pollutants, including annual, seasonal and monthly mean concentrations, was performed. To characterize the North African dust intrusions, the percentage of days with mean values above the defined limit (mean and maximum number of hours per day) was also assessed. The dust plumes observed through the satellite images were delimited, and their areas were calculated. Each plume of the 246 days was delimited and represented seasonally.

A statistical summary was built and analyzed for dust intrusion days (identified by APA and by the study inventory) and for days without dust intrusions with the meteorological and dust concentration variables. With the hospitalization data, an exploratory statistical analysis was also developed.

To evaluate the possible statistical association between emergency hospital admissions and days with dust intrusion, a DLNM (Distributed Lag Nonlinear Model) was applied. This method enables us to perceive the immediate effect of a phenomenon, as well as the lagged and cumulative effect in time (Gasparrini et al. 2010; Gasparrini and Armstrong 2011), where the description of the associations is defined as exposure-lag-response (Gasparrini 2018; Gasparrini et al. 2018).

The production of a DLNM presupposed two phases. Firstly, each variable was assessed individually to identify the combination of degrees of freedom (DF) with the lowest generalized cross-validation (GCV) values. In case of similar GCV values, the parsimony criterion was applied. Then, the most significant combination was chosen to proceed to the next step. In this first phase, the co-variables chosen were the daily average mean temperature and the average daily concentration of PM10 with 2 DF. Secondly, for the final model, two variables were included to adjust for time and seasonality: “Time” and “Days of the week”. The model is represented by the equations [1] and [2].

1 $$ {\uplambda}_i={L}_4\left({\mathrm{temperature}}_i\right)+{L}_4\left(\mathrm{PM}{10}_i\right)\kern0.5em $$

2 $$ {\mu}_i=\mathrm{APA}+{S}_4\left({\mathrm{temperature}}_i\right)+{S}_4\left(\mathrm{PM}{10}_i\right)+{S}_{2\times 11}\left(\mathrm{time}\right)+\mathrm{DOW} $$

where *λ*_*i*_ and *μ*_*i*_ are the urgent hospital admission means for the 2-day lag and prediction model components, respectively; *L*_*k*_(·) and *S*_*k*_(·) are the corresponding space of lag DF and spline nonlinear effects with *k* degrees of freedom. The model was also adjusted to time and days of the week (DOW). These last two variables aim to control possible seasonal and trend variations over the study period that is not related to the hospitalizations.

## Results

### Characterization of dust intrusion events in Central Alentejo

#### Annual, seasonal and monthly distribution

**Fig. 2:**
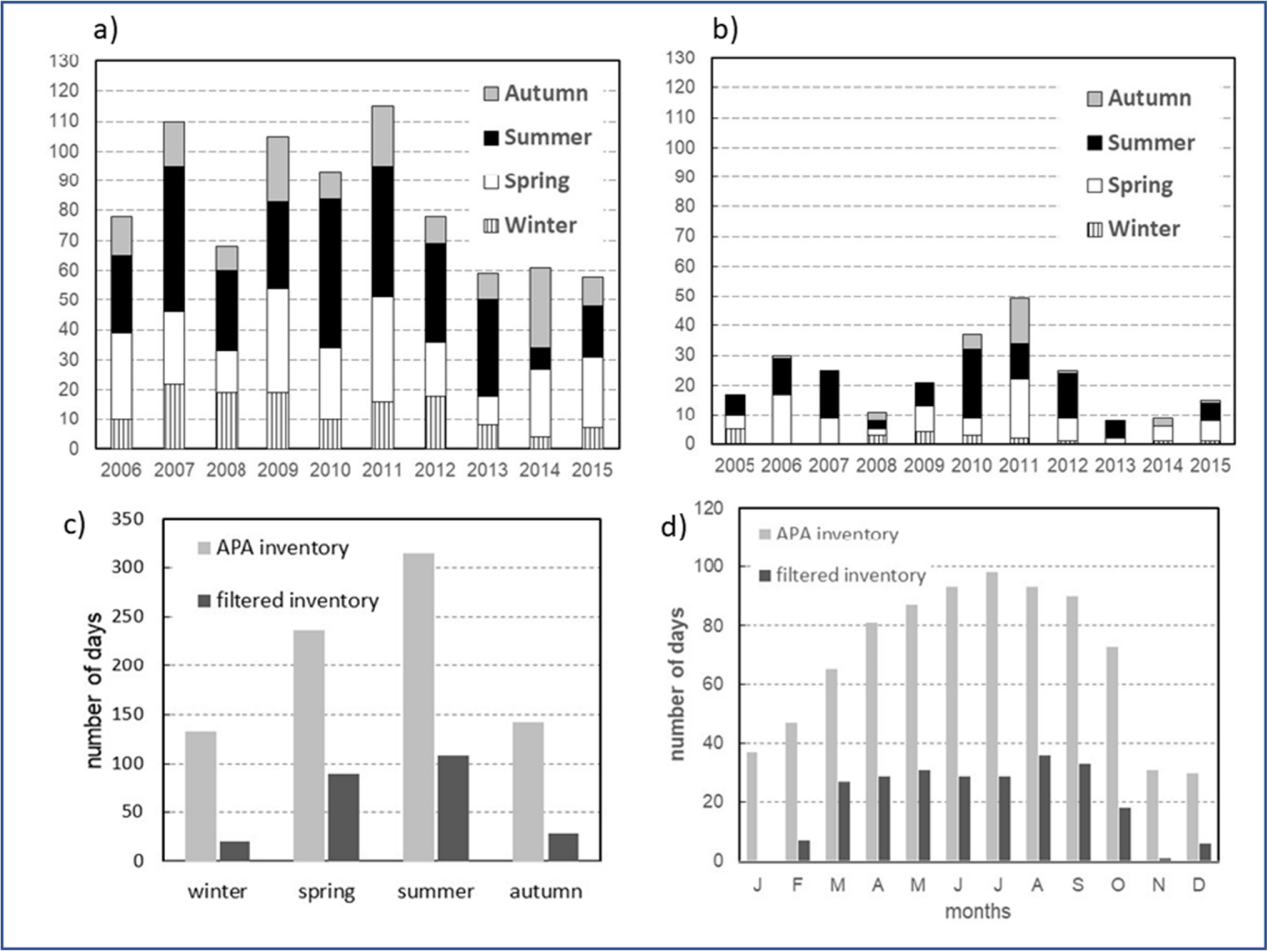
Annual, seasonal and monthly distribution of dust intrusion episodes in Central Alentejo, between 2005 and 2015, according to APA inventory and validation inventory. **a** Annual variation according to APA inventory, **b** annual variation according to validation inventory, **c** seasonal variation, and **d** monthly variation

On an annual scale, according to Fig. 2a, b, a strong interannual variation is visible. In the APA inventory, it can be observed that 2011 had the most episodes while the year with the fewest was 2015. As for the study inventory, the year with the highest number of dust intrusion days was also 2011while 2013 was the year with the lowest frequency. The variation of episodes between seasons and years is irregular, that is, it does not have a pattern that suggests a tendency for growth or decrease over the study period.

Seasonally (Fig. 2c) and monthly (Fig. 2d), both inventories exhibit the same behaviour, where summer and its months have most episodes and winter with the least.

#### Characterization of dust plumes

**Fig. 3:**
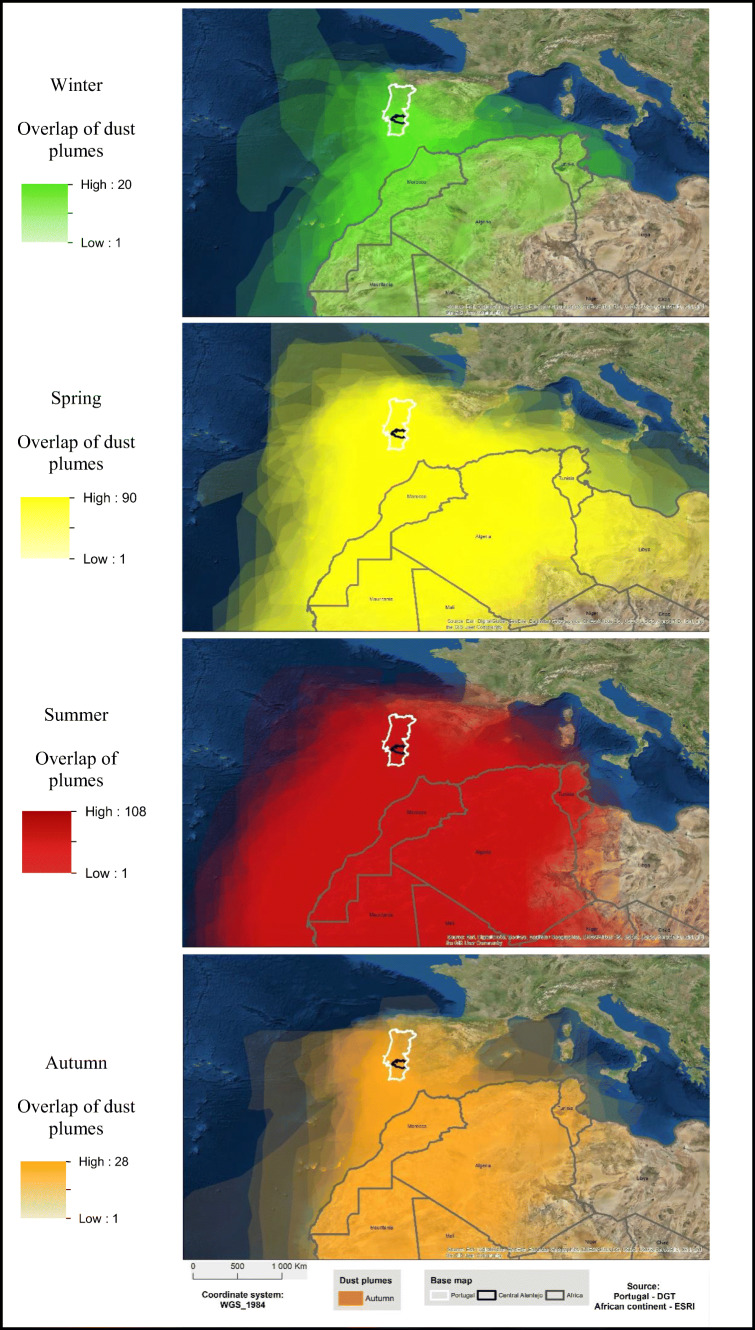
Overlap of dust plumes for each season of the year (2005–2015 period)

During winter (Fig. 3), it is noticed that the concentration and dimension of the plumes are lower. However, an area with a more pronounced hue is visible between the north-west of Morocco and the south of Portugal. It is also observed that the plumes extend from Mauritania, Algeria and Tunisia and can even reach almost the region of the Azores and the Atlantic to the north-west of mainland Portugal. For this reason, through Table 2, it is confirmed by the summed area that the overlap of plumes is low compared to others, resulting from the small number of recorded episodes. This season also has the lowest average and maximum area, revealing that the plumes are spatially limited. In spring (Fig. 3), the situation changes dramatically. In addition to the number of episodes being much higher, the plumes have much larger dimensions (Table 2). It can be seen through Fig. 3 a great overlap of the plumes among the shown area. Through Table 2, it is possible to confirm the idea the figure suggests. Therefore, during the spring season, the summed area increases by almost sixfold compared to winter; however, the average area has only a slight increase. In summer (Fig. 3), the increase in the length of the plumes is even more noticeable. In this season, the overlap of plumes is higher. The plumes thus extend over large areas, not presenting so defined trajectories as those observed in winter, revealing that the plumes are less spatially limited.

**Table 2:** North African dust plume areas (km^2^)

Area	Winter	Spring	Summer	Autumn
Summed	~54,529,660	~295,057,890	~545,999,630	~128,642,970
Mean	~2,726,400	~3,278,420	~5,102,800	~4,435,960
Minimum	~642,040	~238,610	~356,140	~863,940
Maximum	~5,597,220	~8,541,150	~12,387,070	~10,937,500

Based on the data presented in Table 2, it is observed that, in relation to summer, the summed area occupied by the plumes almost doubles compared to spring. The average and maximum areas of each plume increased significantly, while the minimum area increased slightly compared to spring. Finally, during autumn (Fig. 3), the intensity of the plumes and the number of episodes tend to become lower again suggesting some similarities with winter. There is an area with high overlap between Morocco and the south of Portugal, up to the study area roughly. However, the areas of the dust plumes give other indications. According to Table 2, the total area, despite being smaller compared to spring by almost half, the average, minimum and maximum area is higher, that is, autumn has fewer episodes than spring, although these same episodes include larger areas.

#### PM concentrations in dust intrusion events

According to Table 3, only 2 years (2005 and 2008) have more than 50% of the days with the average value above the limit. During 2006, the North African intrusion days had an average of 17 h that the permitted values were exceeded (Table 3). Conversely, in 2009, only 1 h per day on average surpassed the limit. During 2005, 2006 and 2010, at least one of the days had all hourly values above what is allowed by the EU directive for air pollutant concentration. The highest value of PM10 concentration was registered in 2006 (Table 3), and 2013 had the lowest value among the maximum recorded.

**Table 3:** Characterization of mean PM10 concentrations per year, on study inventory days, in Central Alentejo. Data source: QualAr–Terena Rural Background station

Year	% days ≥ 50 μg/m^3^	Daily mean hours ≥ 50 μg/m^3^	Maximum daily number of hours ≥ 50 μg/m^3^	Highest value μg/m^3^
2005	88	16	24	230
2006	37	17	24	640
2007	12	15	18	123
2008	55	13	22	193
2009	29	1	16	151
2010	16	12	24	397
2011	32	12	16	224
2012	32	13	21	250
2013	0	-	9	130
2014	11	13	13	266
2015	20	11	16	219

As per PM2.5 data, only in 2013 half the days exceed the threshold as shown in the supplementary material (Table S2). Regarding the mean and maximum hourly values 2005 and 2010 stand out. The highest recorded value registered was in 2015.

As for the seasonal analysis (Table 4), it is observed that, within the four seasons, less than 50% of the days exceed the designated limits. In any case, winter is the season with the highest percentage of days above the limit, despite having fewer episodes as noted before. The highest value occurs also in the winter. Spring and summer registered days with 24 h exceeding the limit. Spring has the highest value, while autumn has the lowest value.

**Table 4:** Characterization of mean PM10 concentrations per season, on study inventory days, in Central Alentejo. Data source: QualAr–Terena Rural Background station

	% days ≥ 50 μg/m^3^	Daily mean hours ≥ 50 μg/m^3^	Maximum daily, number of hours ≥ 50 μg/m^3^	Highest value μg/m^3^
Winter	45	16	22	244
Spring	30	14	24	640
Summer	32	13	24	397
Autumn	24	11	15	224

### Statistical association between environmental variables and hospital admissions due to respiratory diseases

#### Descriptive analysis

The behaviour of the meteorological and dust concentration variables, according to APA, and the study inventory and the days without dust present slight but important differences as shown in Table 5.

**Table 5:** Statistical summary of independent variables and hospitalizations. Sources: KNMI *Climate Explorer*; ICT - *Atmospheric Sciences Water and Climate*; QualAr; Grouping of Health Centres in Central Alentejo

	Mean	Maximum	Minimum	Standard deviation	*N*
Days without dust					
Mean temperature (°C)	15.9	31.5	2.5	6.1	2635
Maximum temperature (°C)	21.9	42.4	6.2	7.6	2954
Minimum temperature (°C)	9.4	22.2	-4	5.0	2760
Relative humidity %	63.7	98.8	14.7	14.7	3116
Precipitation mm	4.6	39.1	0	7.4	1264
Wind velocity (m/s)	2.1	5.1	0.2	0.8	3116
Mean PM10 concentration (μg/m^3^)	18.0	130.2	0	9.3	2485
Mean PM2.5 concentration (μg/m^3^)	9.6	139	0	6.7	2585
Maximum PM10 concentration (μg/m^3^)	43.4	442	0	31.5	2480
Maximum PM2.5 concentration (μg/m^3^)	26.1	391	0	20.4	2585
Days with dust—APA					
Mean temperature (°C)	20.7	33.4	7.05	5.8	722
Maximum temperature (°C)	28.0	42.4	8,6	7.7	790
Minimum temperature (°C)	13.0	24.7	1	4.5	742
Relative humidity %	55.5	91	15.7	14.5	812
Precipitation mm	4.6	32.5	0	6.8	355
Wind velocity (m/s)	1.8	3.8	0.6	0.6	822
Mean PM10 concentration (μg/m^3^)	33.7	155.6	4	15.9	662
Mean PM2.5 concentration (μg/m^3^)	14.2	38.9	0	5.5	693
Maximum PM10 concentration (μg/m^3^)	72.6	640	16	49.9	657
Maximum PM2.5 concentration (μg/m^3^)	34.6	222	0	22.8	693
Days with dust study inventory					
Mean temperature (°C)	22.4	31.45	8.45	5.5	221
Maximum temperature (°C)	30.1	41.8	12.6	7.0	236
Minimum temperature (°C)	14.6	23.6	2.3	4.3	228
Relative humidity %	54.1	90.9	24.3	13.5	239
Precipitation mm	2.8	16.8	0	4.2	112
Wind velocity (m/s)	1.7	3.4	0.9	0.5	240
Mean PM10 concentration (μg/m^3^)	47.6	155.6	4	22.7	193
Mean PM2.5 concentration (μg/m^3^)	16.1	35.0	0	6.0	194
Maximum PM10 concentration (μg/m^3^)	103.6	640	16	70.4	193
Maximum PM2.5 concentration (μg/m^3^)	41.1	222	0	31.7	194

The mean, maximum and minimum average temperatures present the lowest values during days without dust (15.9°C, 21.9°C and 9.4°C, respectively). In contrast, the study inventory days have the highest values (22.4°C, 30.1°C and 14.6°C, respectively). It should also be noted that the values on APA days are higher than on days without dust. Regarding relative humidity, this variable shows higher values on days without dust (63.7%) and lower on days with dust (55.5–54.1%). The precipitation and the wind speed do not show significant variations depending on the presence/absence of dust, although on study inventory days, the values are slightly lower. As for the set of variables of the PM10 and PM2.5, the concentration is higher on dusty days, as would be expected.

**Fig. 4:**
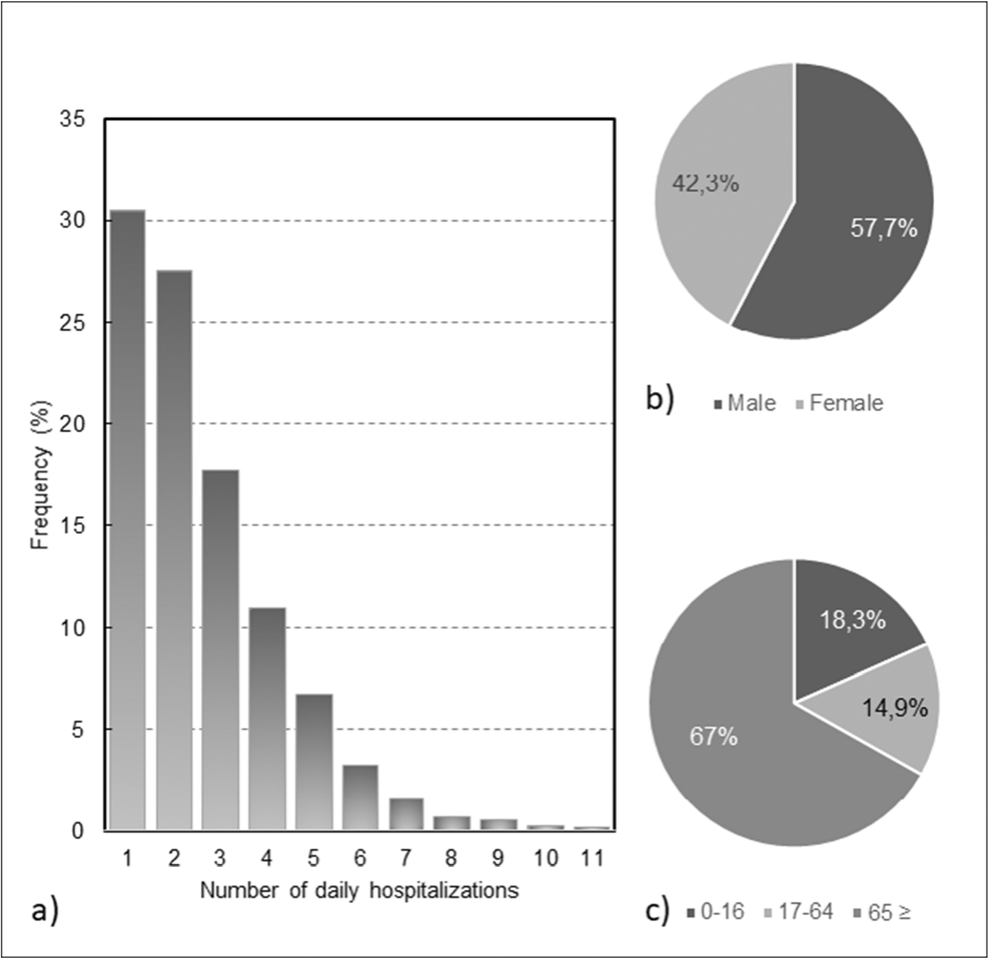
Hospitalizations in Central Alentejo (2005–2015): **a**) frequency of hospitalizations per day due to respiratory diseases, **b**) hospitalizations by gender, and **c**) hospitalizations by group age

**Fig. 5:**
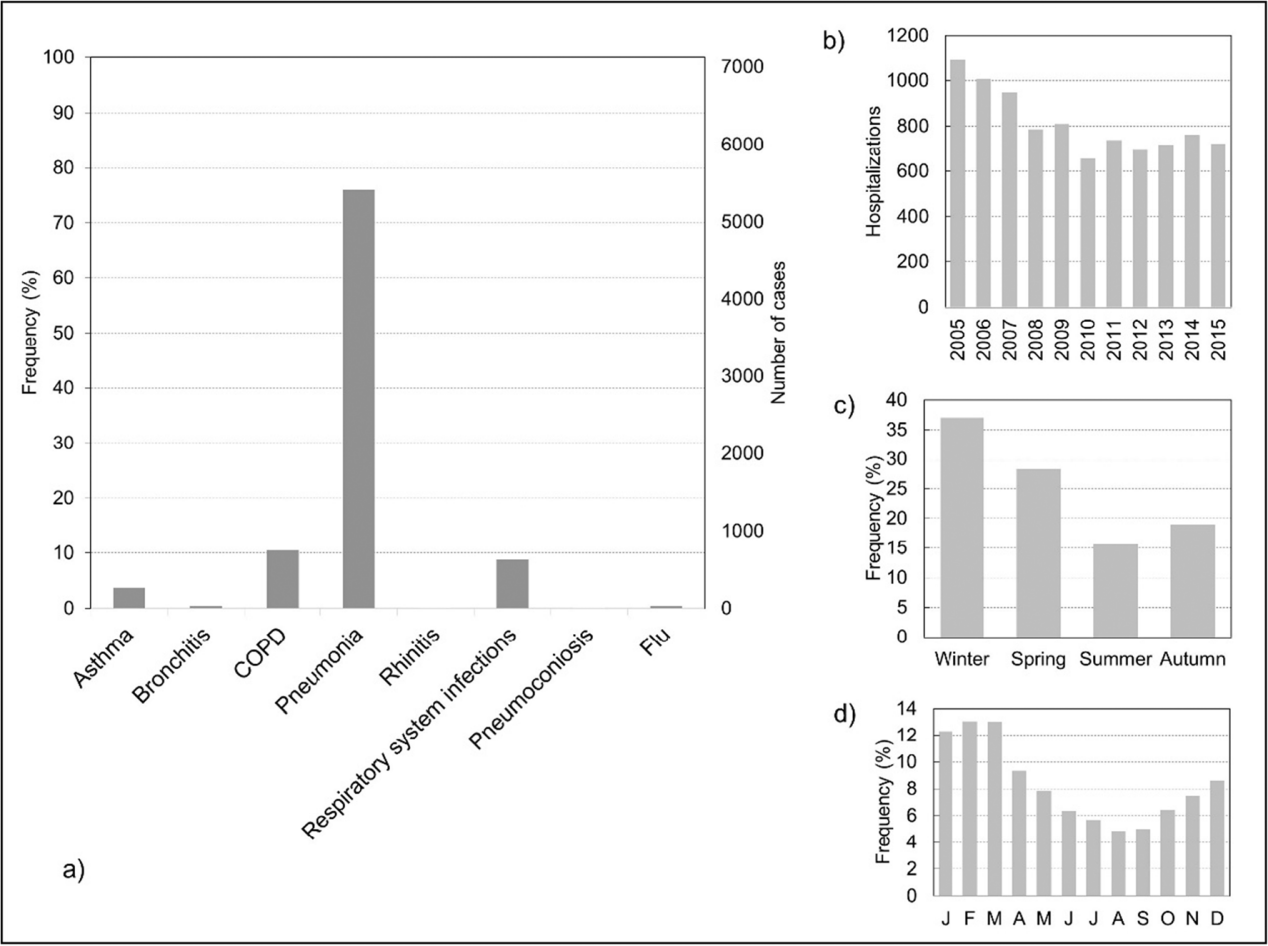
Hospitalizations in Central Alentejo (2005–2015): **a**) total number of hospitalizations per respiratory disease, **b**) hospitalizations by year, **c**) hospitalizations by season, and **d**) hospitalizations by month

In Central Alentejo, between 2005 and 2015, 7123 individuals were hospitalized in a total of 8933 urgent hospitalizations (several individuals with more than one hospitalization), due to respiratory diseases. In 30.5% of the days, one hospitalization per day was observed while, in contrast, in 13.1% of the days there were five or more hospitalizations (Fig. 4). Of the total number of urgent admissions, 42.3% were of women and 57.7% were of men (Fig. 4). The elderly are the most vulnerable age group (Fig. 4). Considering all urgent admissions, it is also observed that most of them were mainly due to pneumonia (Fig. 5a).

It is observed that hospitalizations due to respiratory diseases (Fig. 5b) show a pattern of decrease. Probably due to the influence of cold weather on health, the winter registered the highest number of hospitalizations, while the summer the least (Fig. 5c). The monthly analysis (Fig. 5d) shows that during winter months, there is a higher percentage of people hospitalized with respiratory problems, with February being the most problematic. In contrast, the summer months confirm that during this season, there are less hospitalizations, with August being the month with less hospitalizations (4.5%).

#### DLNM modelling of hospitalizations due to respiratory diseases and environmental determinants

**Fig. 6:**
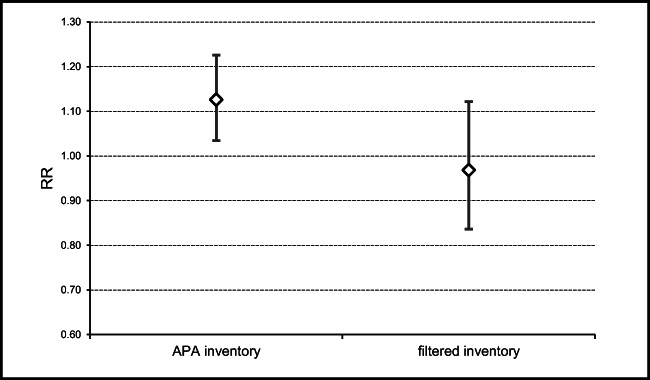
Boxplot of the relative risk of urgent hospitalizations due to the intrusion of dust in Central Alentejo, according to the study inventories, with confidence intervals

The result obtained with the DLNM (Fig. 6) shows that the relative risk of urgent hospitalizations due to respiratory diseases is 12.6% (*p*-value <0.05) higher on dust intrusion days identified by APA after the model being adjusted for the average daily temperature, average daily PM10, time and day of the week. The study inventory was inserted as a variable only to be as a mean of comparison with APA inventory in order to verify if with a stricter inventory a significantly different result would be found. Despite the association between dust intrusion days and hospital admissions is not significant (*p*-value > 0.05) when considering the study inventory, it does not mean that the identified dust intrusions do not have a hazardous effect on human health. It needs to be highlighted that the dust intrusion days from the study inventory are not significant in the model; however, dust days from APA inventory are.

## Discussion

In Central Alentejo, from 2005 to 2015, it was verified through the DLNM that there is a significant statistical association between urgent hospital admissions due to respiratory afflictions and dust intrusions. This statistical relationship means that after adjustment with the daily average mean temperature and daily average PM10, the days with dust intrusion, identified by APA, have a relative risk of 12.6% more urgent hospitalizations than on days without dust intrusions. Part of the explanation for urgent hospital admissions for respiratory diseases in Central Alentejo is conditioned by these variables. However, important contributing factors to hospitalizations were not modelled in this study, such as individual health behaviour, lifestyle and physiological characteristics. Therefore, this result is liable to change, and further studies are needed. Nonetheless, the results highlight the role of dust intrusions as an important health determinant for respiratory diseases in Alentejo Central.

The results of studies carried out in several countries and cities in the South of Europe (namely Spain, Italy, Greece and Cyprus) by authors such as Alessandrini et al. (2013), Díaz et al. (2012), Jiménez et al. (2010), Mallone et al. (2011), Middleton et al. (2008), Neophytou et al. (2013), Pérez et al. (2008, 2012), Reyes et al. (2014), Sajani et al. (2011), Samoli et al. (2011a, 2011b), Stafoggia et al. (2016) and Tobías et al. (2011) are aligned with ours and demonstrate that Saharan dust intrusion events have a statistical association with an increase in hospitalizations and mortality from cardiac, respiratory and even cerebrovascular causes, particularly among the elderly age group.

Through the statistical summary, it is also noticed that generally in the Évora’s hospital, there is in most days an urgent hospitalization per day due to respiratory pathologies. On the other hand, it was also found that men register more hospitalizations than women. As for the age group, the elderly (65 years old or more) are more vulnerable than young people (less than 17 years old) as shown in the results. It is also mentioned by Griffin and Kellogg (2004), Jiménez et al. (2010), Sajani et al. (2011) and Samoli et al. 2011a, 2011b) that the elderly age group is the most affected. The urgent hospitalizations are commonly due to pneumonia. The impact of dust intrusions in the increase of these diseases have already been mentioned by Goudie (2014) and UNEP et al. (2016) in the case of pneumonia and by Brunekreef and Holgate (2002), Chien et al. (2014), Goudie and Middleton (2006) and Middleton (2017) in the case of obstructive pulmonary diseases. On the other hand, the less common diseases that cause urgent hospitalizations are rhinitis, pneumoconiosis and infections in the upper part of the respiratory system. The influence of dust events on rhinitis and respiratory system infections was identified by Goudie (2014) and UNEP et al. (2016).

According to the data analyzed, during winter there are more hospitalizations compared to other seasons, although the emission and intrusion of dust into the atmosphere are lower (Cachorro et al. 2006; Pey et al. 2013; Querol et al. 2010). This might be explained by some factors such as cold temperatures and energy poverty. Some studies such as Almendra et al. (2019a), Almendra et al. (2019b), Silva (2015) and Vasconcelos et al. (2013) focused on this association and found important results. The emission of dust during the winter is associated with very severe storms with the presence of cumulonimbus and vertical movements in the atmosphere (Goudie and Middleton 2006). Therefore, winter’s episodes affecting the Iberian Peninsula (Querol et al. 2010) are infrequent and do not last very long (Cachorro et al. 2006; Pey et al. 2013) and yet, with plumes with higher concentrations compared to other seasons (Cachorro et al. 2006; Pey et al. 2013). Within winter, the least active period is November and December (Escudero et al. 2005; Knippertz and Todd 2012). In fact, the results confirm exactly this situation, in which the winter is the season where fewer episodes are observed in the Central Alentejo. Within this period, the months with the least activity are November, December and January. The present study also confirmed that the frequency and dimension of the plumes in the set of episodes during the winter months are lower, with plumes of dust that, in terms of average and maximum area, have lower values compared to other seasons. Despite this, the PM10 daily average concentration indicates that, during winter, the legal limit is exceeded in 45% of the days. Although the objective of this work is not to explain this phenomenon, we can present some factors that may help to explain it, for example, there is a higher concentration of particles during the winter, because the area occupied by the plumes are more restricted; it may also be explained by other factors such as atmospheric boundary layer height.

The spring is the second season with the most episodes and urgent hospitalizations. In terms of its months, according to the APA inventory, June is part of the top 3 months with the most episodes. According to the study inventory, there are 2 months in this season that are part of the top three with more episodes, April and June. In fact, as suggested by Escudero et al. (2005), the months when more dust intrusions are observed are between May and August. Knippertz and Todd (2012) mention September as the limit instead of August. Escudero et al. (2005) state that April is a month with very few episodes, although in the study inventory, this month was well represented. As for urgent hospitalizations during this season, April registered the highest value. As for the dust plumes identified in this season, they increased in number and their dimensions are also larger. Cachorro et al. (2006) state that during spring, the dust plumes begin to be more extensive and diluted (not as much as in the summer). This argument is validated through the plume areas shown. Thus, they covered 6 times more area than in winter, with the average plume area increasing by about one million square kilometres. As for the concentration of PM10 particles, only 30% of the days recorded in this period had daily average values above the permitted, while PM2.5 had only 9%.

As for hospitalizations in the summer, there are fewer hospitalizations. However, it is during summer that the greatest number of episodes occurred (Prospero 1996; Goudie and Middleton 2001; Engelstaedter et al. 2006; Marconi et al. 2014), which are due to the strong emissions of particles into the atmosphere caused by movement of the Intertropical Convergence Zone to the north, which allows the injection of dust in the free atmosphere and which is later transported with greater intensity to the Iberian Peninsula, the Mediterranean and also to the Atlantic Ocean (Querol et al. 2010). Summer is, in fact, the season with most episodes, according to both inventories. Regarding the months, the most active are between May and August (Escudero et al. 2005) or between May and September (Knippertz and Todd 2012). According to the APA inventory, the month with the most episodes is July, followed by August, while according to the study inventory, the most active month is August, followed by September. As for hospitalizations in these months, it is noted that there are fewer hospitalizations in August. The overlap of plumes during the summer is quite noticeable, due to the large number of episodes, doubling its occupied area compared to spring. The average, maximum and minimum areas of the plumes increased in summer. According to Cachorro et al. (2006) and similarly to what was referred to for spring, during this season, there are very extensive and less concentrated plumes. Regarding the concentration of PM10 particles, only 32% of the study inventory days had daily averages above the limit. August registers half of the days above the limit. As for PM2.5, the percentage of days in excess of the legal limit value is very low.

As for the hospitalizations during autumn, it is the second season with fewer hospitalizations. As for the emission of dust at this time of year, it starts to become less intense, especially in late autumn, which is already weak (Querol et al. 2010). Thus, the results of the present investigation demonstrate that the autumn is the third season with most episodes which is in agreement with Cachorro et al. (2006). The number of episodes during this period is mainly distributed between September and October (during the autumn days), decreasing to very low values, afterwards. The number of hospitalizations is higher in November, with signs of an increase as winter approaches. As for the plumes, they are more intense but in less quantity (Cachorro et al. 2006; Pey et al. 2013). This is confirmed by the plumes, representing fewer episodes. However, their areas give other indications considering that the medium, minimum and maximum areas show that they individually occupy larger areas. As for the particle’s concentrations, autumn has the lowest percentage. Regarding the frequency of episodes over the years of analysis, there was no visible trend of either increasing or decreasing, although Goudie and Middleton (2006) mention that North Africa has shown a trend of increasing particle emissions for the atmosphere.

## Conclusion

The relative risk of urgent hospitalizations due to respiratory diseases in Central Alentejo is 12.6% higher during Saharan dust intrusion days. Despite the limitations of this study, the results show the role of dust intrusions as an important health determinant for respiratory diseases in Alentejo highlighting the public health need to address this factor. It has also been found that there are more hospitalizations in winter. This can be explained by climatic conditions, namely the temperature, and other factors such as individual health behaviour, lifestyle, human physiology, higher levels of pollution or energy poverty. Thus, although there are more hospitalizations in winter, it also has fewer episodes of dust intrusion. Despite the smaller number of episodes, in 45% of them, an average daily value of PM10 surpassed the limit value imposed by the EU directive. Thus, in winter, dust may influence urgent hospitalizations by worsening air quality and aggravating prevalent respiratory pathologies. During summer, the opposite was shown. There are more intrusion events, although the percentage of days with mean PM10 above the limit was lower. One of the reasons for this may be due to the bigger extension of dust plumes, hence less concentration of particles.

This study aims to boost other studies regarding the occurrence of these phenomena in Portugal and their effects on human health, namely in terms of hospitalizations or mortality, since in other southern European countries, this research topic is much more developed.

To conclude, it must be stressed that it is very important to continue to monitor, predict and study the occurrence of Saharan dust intrusions in Portugal, to prevent the harmful effects of air quality deterioration. This is particularly relevant to ensure that the population, especially the most vulnerable age groups (>65 years), are prepared for the occurrence of these events. It is imperative that studies in Portugal on this subject should be further developed, not only on its effects on health and mortality, but also on soils and agriculture, in order to mitigate the human and the environmental impacts of dust intrusions.

## Supplementary Information

ESM 1 (DOCX 32 kb)
