# Supplementary material for: North African dust intrusions and increased risk of respiratory diseases in Southern Portugal
Source: Int J Biometeorol. 2021 Apr 22;65(10):1767–80. doi: 10.1007/s00484-021-02132-x (PMC8437926; doi:10.1007/s00484-021-02132-x)
Supplement: Supplementary file 1 — (DOCX 32 kb) [file 484_2021_2132_MOESM1_ESM.docx]

Title:

**North African dust intrusions and increased risk**

**of respiratory diseases in Southern Portugal**

Authors:

Silva, T.; Fragoso, M.; Almendra, R.; Vasconcelos, J.; Lopes, A.; Faleh, A.

To be submitted to

*International Journal of Biometeorology*

April 2020

**Electronic supplementary material**

**Table S1:** ICD-9-CM code of the diseases chosen for this study. **Data source:** GHCCA. Self-elaboration.

| **ICD-9-CM code** | | | | | | | |
| --- | --- | --- | --- | --- | --- | --- | --- |
| **Asthma** | **Bronchitis** | **Chronic obstructive pulmonary disease** | **Pneumonia** | | **Infections in the lower part of the respiratory system** | **Infections in the upper part of the respiratory system** | **Rhinitis** |
| 49300 | 490 | 49120 | 4800 | 48239 | 46611 | 4658 | 4720 |
| 49301 | 4910 | 49121 | 4801 | 48240 | 46619 | 4659 | **Pneumoconiosis** |
| 49302 | 4911 | 49122 | 4808 | 48241 |  | 4789 | 502 |
| 49310 | 4918 |  | 4809 | 48242 |  |  | 505 |
| 49312 |  |  | 481 | 48249 |  |  | **Flu** |
| 49321 |  |  | 4820 | 48281 |  |  | 4870 |
| 49322 |  |  | 4821 | 48282 |  |  | 4871 |
| 49391 |  |  | 4822 | 48283 |  |  | 4878 |
| 49392 |  |  | 48230 | 48289 |  |  |  |
|  |  |  | 4830 | 4829 |  |  |  |
|  |  |  | 4838 | 486 |  |  |  |

**Table S2**: Characterization of mean PM2.5 concentrations per year, on validation days, in Central Alentejo.

**Data source:** QualAr – Terena Rural Background station.

|  | **% days ≥ 50 µg/m^3^** | **Daily mean hours ≥ 50 µg/m^3^** | **Maximum daily. number of hours ≥ 50 µg/m^3^** | **Highest value µg/m^3^** |
| --- | --- | --- | --- | --- |
| **2005** | 6 | 17 | 21 | 87 |
| **2006** | 3 | 11 | 11 | 85 |
| **2007** | 32 | 11 | 16 | 123 |
| **2008** | 0 | - | - | - |
| **2009** | 14 | 12 | 14 | 93 |
| **2010** | 5 | 17 | 21 | 48 |
| **2011** | 0 | - | - | - |
| **2012** | 0 | - | - | - |
| **2013** | 50 | 9 | 12 | 222 |
| **2014** | 0 | - | - | - |
| **2015** | 13 | - | 11 | 229 |

**Table S3**: Characterization of mean PM2.5 concentrations per season, on validation days, in Central Alentejo.

**Data source**: QualAr – Terena Rural Background station.

|  | **% days ≥ 50 µg/m^3^** | **Daily mean hours ≥ 50 µg/m^3^** | **Maximum dailynumber of hours ≥ 50 µg/m^3^** | **Highest value µg/m^3^** |
| --- | --- | --- | --- | --- |
| **Winter** | 5 | 12 | 12 | 44 |
| **Spring** | 9 | 11 | 21 | 94 |
| **Summer** | 10 | 12 | 21 | 222 |
| **Autumn** | 3 | 11 | 11 | 131 |
